# Editorial for the Special Issue on GaN-Based Materials and Devices: Research and Applications

**DOI:** 10.3390/mi16060652

**Published:** 2025-05-29

**Authors:** Wei Liu, Kun Wang

**Affiliations:** School of Microelectronics, Northwestern Polytechnical University, Xi’an 710072, China; kunwang@nwpu.edu.cn

Gallium nitride (GaN)-based materials and devices, as a core representative of third-generation semiconductors, have emerged as a strategic frontier driving modern electronics and optoelectronics revolutions [1]. Owing to its wide bandgap, high breakdown field, and superior electron mobility, GaN demonstrates transformative potential in power devices [2,3,4], radio-frequency communications [5,6], and optoelectronic applications [7,8]. However, to address stringent demands for high efficiency, high power density, and miniaturization, continuous advancements in GaN material optimization and device engineering remain imperative.

Cross-disciplinary integration of microelectronics, materials science, and photonics provides fundamental support for optimizing GaN-based technologies. For instance, the microelectronics theory underpins device structure design and carrier transport optimization, the materials science focuses on enhancing crystal quality and cost-effective manufacturing, and the photonic principles contribute to photon generation, manipulation, and detection, enabling breakthroughs in laser diodes and photodetectors. Recent innovations, including heterojunction engineering [9], epitaxial growth optimization [5], graded structures [10], and superlattice buffer layers [11], have collectively driven the evolution of GaN-based materials and devices toward compactness and enhanced performance. Therefore, with the aim of highlighting the cutting-edge research breakthroughs, this Special Issue presents a diverse collection of recent advances in GaN-based materials and devices, offering valuable insights and practical methodologies that will undoubtedly be of great benefit to researchers in the field.

In total, there are five articles included in this Special Issue, each focusing on different aspects of GaN-based materials and devices. Sun et al. innovatively employed LPCVD SiNx as both a gate dielectric and surface passivation layer for AlGaN/GaN MISHEMTs, exploring how growth parameters such as temperature, pressure, and gas flow ratio influence SiNx quality and its impact on 2DEG density and interface trap density [12]. This work advances the understanding of SiNx passivation in high-power devices, offering practical insights for improving device performance. Another article written by Xu et al. presents a high-power GaN lateral photoconductive semiconductor switch (PCSS) device, achieving an impressive output peak current of 142.2 A with an input voltage of 10.28 kV [13]. This study introduces a novel method of retaining the AlGaN/GaN heterostructure between electrodes, which effectively enhances the output peak current of the PCSS, offering a practical approach to improve device performance. Ji-Hun Kim et al. explored the operational characteristics of AlGaN/GaN HEMTs using various high-k passivation materials (Al_2_O_3_ and HfO_2_) and different passivation structures to enhance breakdown voltage while mitigating the reduction in cut-off frequency [14]. Their work presents a novel approach to optimizing passivation structures for high-power and high-frequency applications, addressing the critical trade-off between voltage and frequency characteristics in AlGaN/GaN HEMTs. In another work, Chen et al. investigated the degradation behaviors of ferroelectric gate GaN HEMTs under positive gate bias stress, comparing devices with pure PZT and composite PZT/Al_2_O_3_ gate oxides [15]. This research provides novel insights into the reliability challenges of ferroelectric gate GaN HEMTs, suggesting they may face more severe degradation than conventional MIS-HEMTs, which is crucial for the development of robust high-power and high-frequency devices. Finally, Liu et al. investigated the electroluminescence characteristics of yellow-light-emitting InGaN/GaN multiple quantum wells (MQWs) with varying indium (In) content in the last quantum well, providing insights into the physical mechanisms affecting carrier capture and light emission [16]. Their research work offers a novel understanding of optimizing In content distribution to improve the efficiency of longer-wavelength InGaN-based light-emitting devices, addressing a critical challenge in the field.

As the global GaN market is expected to hit USD 60 billion by 2030, its technological advancements are set to accelerate cross-domain innovation across renewable energy, biomedicine, and advanced display systems. This Special Issue spotlights the latest research and emerging applications of GaN-based materials and devices, delivering breakthrough insights and engineering solutions that will be essential references for researchers aiming to overcome technological challenges and broaden application scopes.
